# Effect of Digital Treadmill on Lower Limb Motor Function in Patients with Ischemic Stroke

**DOI:** 10.12669/pjms.41.3.9572

**Published:** 2025-03

**Authors:** Yuqing Huang, Ning Wang, Xincai Yang, Yun Deng, Shuai Yan

**Affiliations:** 1 Yuqing Huang, Guizhou Center for Disease Control and Prevention, Guiyang 550000, Guizhou, China. Department of Rehabilitation Medicine, Affiliated Hospital of Hebei University, Baoding 071000, Hebei, China; 2 Ning Wang, Department of Rehabilitation Medicine, Affiliated Hospital of Hebei University, Baoding 071000, Hebei, China; 3 Xincai Yang, Department of Rehabilitation Medicine, Affiliated Hospital of Hebei University, Baoding 071000, Hebei, China; 4 Yun Deng, Department of Rehabilitation Medicine, Affiliated Hospital of Hebei University, Baoding 071000, Hebei, China; 5 Shuai Yan, Department of Neurological Function Examination, Department of Rehabilitation Medicine, Affiliated Hospital of Hebei University, Baoding 071000, Hebei, China

**Keywords:** Balance Function, Digital Treadmill, Gait Analysis, Hemiplegia, Rehabilitation

## Abstract

**Objective:**

To observe the effect of digital treadmill on lower limb motor function in patients with ischemic stroke.

**Method:**

This was a clinical comparative study. A total of 60 patients with ischemic stroke at Hebei University Hospital were selected from October 2020 to September 2022 and randomly divided observation group and control group. The control group was given conventional rehabilitation therapy, with the observation group receiving digital treadmill training. Both groups were assessed using the Holden Functional Ambulation Category Scale(FAC), digital treadmill gait analysis system, and dynamic balance instrument and after four weeks, followed by a statistical analysis.

**Result:**

After four weeks of treatment, both groups showed an improved in FAC grading, gait spatiotemporal parameters (including step speed, step length, stance time and step height), gait kinematic parameters (including hip joint flexion and extension mobility, knee joint flexion and extension mobility, and active ankle dorsiflexion ability) and balance assessment parameters (including motion trajectory area and motion trajectory perimeter) compared to pre-treatment (p< 0.05). Additionally, the gait symmetry parameters, including step length deviation and step height deviation, in the observation group were significantly lower than pre-treatment (p< 0.05), while in the control group, without statistically significant differences(p>0.05). Furthermore, the inter-group comparisons showed significant improvements of all the parameters studied above in the observation group compared to the control group (p< 0.05).

**Conclusion:**

Digital treadmill training may effectively improve gait parameters, gait symmetry, walking and balance ability of patients with ischemic stroke.

## INTRODUCTION

Stroke is a type of hemorrhagic or ischemic brain injury accompanied by motor disfunction, sensory changes, cognitive impairment, speech disorders, or coma, and it has long been a significant global health issue.^1^ Walking ability is a major determinant of life quality for stroke patients, so the recovery of walking function has always been a critical part of rehabilitation. The posture of a person during walking is called gait, with symmetry as one of its important features, reflecting the similarity of spatiotemporal, kinematic, or dynamic parameters between the left and right lower limbs. Impaired balance control is deemed a potential factor causing gait asymmetry, which has not received enough attention.^2^ Studies have shown that balance contributes to improving gait speed and symmetry.^3^

There are various treatment methods to improve motor impairments, such as exercise therapy, occupational therapy, medium-frequency pulse electrotherapy, electromyogram(EMG) biofeedback training, etc. However, it is difficult to achieve the desired results with the above methods. In contrast, treadmill walking is a continuous and relatively symmetrical gait training method with a modern analysis system, transforming the original simple qualitative description into quantitative analysis.^4^,^5^ In recent years, a large number of studies have reported on the efficacy of conventional rehabilitation therapy for lower limb motor function in stroke patients^6^, but there are few studies on digital treadmill training. This study was designed to further investigate the impact of digital treadmill training on the gait and balance abilities of hemiplegic patients.

## METHODS

This was a clinical comparative study. Sixty patients with ischemic stroke admitted to Affiliated Hospital of Hebei University from October 2020 to September 2022 were randomly divided into control group(n=30) and observation group(n=30). The control group was given standardized internal medicine treatment and conventional rehabilitation treatment, and the observation group was given digital treadmill training on the basis of the control group’s treatment.

### Ethical Approval:

The study was approved by the Institutional Ethics Committee of Affiliated Hospital of Hebei University (No.: HDFYLL-KY-2024-007; Dated: January 27, 2024), and written informed consent was obtained from all participants and their families.

### Inclusion criteria:

- First-time stroke patients with unilateral hemiplegia, Brunnstrom stage ≥ IV, and FAC score ≥ 2.
- Aged 45-65 years, with a disease course of four-six weeks.
- Patients who could pass the one-minute walking ability assessment of the digital treadmill.
- Patients with stable condition and vital signs and cooperate with treatment and rehabilitation assessment.

### Exclusion criteria:

- Patients with severe internal medical conditions, unstable vital signs, or bleeding tendencies.
- Patients with severe cognitive impairment and mental disorders (MMSE ≤ 9 points).
- Patients with bilateral limb paralysis or limb disability.
- Patients with severe limb pathology, such as severe lumbar spine disease, osteoarthritis, fractures, diabetic foot, etc.
- Patients with newly developed lower limb venous thrombosis.

Conventional rehabilitation therapy included exercise, occupational, medium-frequency pulse electrical stimulation and EMG biofeedback therapy. Exercise and occupational therapy were each treated for 30 minutes, once a day, six days a week; medium-frequency pulse electrical stimulation and EMG biofeedback therapy were each administered for 20 minutes, once daily, six days per week. The digital treadmill training was performed using the Italy-made Walker View Performance 1.0 system. The display screen could record and display relevant real-time data. As the patient’s training and walking ability improved, the slope and speed of the treadmill were gradually increased. It lasted for 20 minutes, once daily, six days per week, for a total of four weeks. The two groups of patients were followed up for six months.

After four weeks of treatment, the lower limb motor function of patients in both groups was assessed using the Functional Ambulation Category Scale (FAC). The Walker View 1.0 was used to assess the spatiotemporal and kinematic parameters of the patients. Walking speed, ankle active dorsiflexion joint mobility were measured manually. The PK252 balance testing system was utilized to assess the balance ability. The area and perimeter of the body’s center of gravity movement trajectory were obtained.

### Statistical Analysis:

SPSS 21.0 software was used for the statistical analysis, with a significance level of α=0.05. Measurement data were presented as mean ± standard deviation(x±s), while categorical data were presented as number of cases and percentages (%). If the data conformed to normal distribution, inter-group comparisons of data were analyzed using independent samples t-test and intra-group comparisons of pre-and post-treatment using paired samples t-test. However, if not, the Mann-Whitney U nonparametric rank-sum test was used for inter-group dates and Wilcoxon test for intra-group comparisons. Comparisons of categorical data were conducted using the chi-squared test. Ordinal data were analyzed by using the Mann-Whitney U non-parametric rank-sum test.

## RESULTS

There were no statistically significant differences in baseline date including gender, hemiplegic side, age, and course of disease between the two groups(p> 0.05) Table-I. No significant difference was found in FAC levels between the observation and control groups pre-treatment(p> 0.05). After four weeks, there were significant differences in FAC levels between pre-and post-treatment of each group (p< 0.05), and post-treatment of the two groups (p< 0.05) Table-II.

**Table-I:** Comparison of General Profile between the 2 Groups.

Group	n	Gender		Hemiplegic Side		Age (years)	Course of Disease (Week)
		Female	Male	Left	Right		
Observation	30	11(36.7)	19(63.3)	15(50.0)	15(50.0)	56.967±3.864	5.000±0.587
Control	30	14(46.7)	16(53.3)	18(60.0)	12(40.0)	55.200±4.046	5.167±0.648
*P*		0.432		0.436		0.089	0.541

**Table-II:** Comparison of Pre-and Post-Treatment FAC Levels between the 2 Groups.

Group	Pre-treatment				Post-treatment				Z	p

	Level 2	Level 3	Level 4	Level 5	Level 2	Level 3	Level 4	Level 5		
Observation	8(26.7)	18(60.0)	4(13.3)	0	0	5(16.7)	20(66.7)	5(16.7)	-4.379	0.000**
Control	5(16.7)	21(70.0)	4(13.3)	0	0	11(36.7)	18(60.0)	1(3.3)	-4.919	0.000**
P	0.495				0.030*					

No significant differences were found in the four indicators of step speed, step length of the affected side, stance time, and step height between pre-treatment of the two groups (p> 0.05); while significant differences arose in all the four indicators between pre-and post-treatment of each group and post-treatment of the two groups after four weeks (p< 0.05). The observation group had higher step length, step speed, stance time and lower step height than the control group (p< 0.05), the same as post-treatment than pre-treatment of each group Table-III.

**Table-III:** Comparison of Pre-and Post-Treatment Spatiotemporal Parameters of Affected Side between the 2 Groups.

Indicator	Group	Pre-treatment	Post-treatment	P
Step length (cm)	Observation	18.567 ± 4.561	31.933 ± 4.417	0.000**
	Control	19.100 ± 5.416	27.367 ± 4.810	0.000**
	P	0.681	0.000^*^	
Step speed (m/s)	Observation	43.877 ± 10.983	57.608 ± 13.908	0.000**
	Control	43.504 ± 5.269	51.062 ± 8.017	0.000**
	P	0.867	0.030^*^	
Stance time (s)	Observation	1.919 ± 1.243	2.708 ±1.138	0.000**
	Control	1.835 ± 0.610	2.072 ± 0.482	0.044**
	P	0.740	0.003^*^	
Step height (cm)	Observation	8.700 ± 2.781	6.233 ± 1.431	0.000**
	Control	8.567 ± 2.359	7.333 ± 2.644	0.007**
	P	0.842	0.049^*^	

***Note:*** Inter-comparison between the observation and control groups shows statistical significance (*p< 0.05). Intra-comparison in the observation and control groups shows statistical significance (**p< 0.05).

There were no significant differences in hip flexion and extension mobility, knee flexion and extension mobility, and active ankle dorsiflexion mobility between the two groups before treatment, (p> 0.05). After four weeks of treatment, the above factors of the post-treatment were higher than those of pre-treatment and the observation group had better improvement than the control group, with statistically significant differences (p< 0.05) Table-IV. At the start of the study, there were no significant differences in step length deviation, step height deviation, ratio of hip flexion and extension mobility and ratio of knee flexion and extension mobility of the affected and unaffected sides between the two groups (p> 0.05).

**Table-IV:** Comparison of Pre-and Post-Treatment Kinematic Parameters of Affected Side between the 2 Groups.

Indicator	Group	Pre-treatment	Post-treatment	p
Hip flexion and extension mobility	Observation	23.297 ± 5.646	30.320 ± 4.950	0.000**
	Control	23.273 ± 3.321	26.443 ± 3.328	0.000**
	P	0.985	0.001^*^	
Knee flexion and extension mobility	Observation	25.717 ± 13.379	35.850 ± 12.346	0.000**
	Control	24.700 ± 6.892	29.703 ± 5.038	0.000**
	P	0.713	0.016^*^	
Active ankle dorsiflextion mobility	Observation	1.733 ± 6.231	7.667 ± 5.320	0.000**
	Control	1.833 ± 6.727	4.333 ± 5.486	0.000**
	P	0.953	0.020^*^	

***Note:*** Inter-comparison between the observation and control groups shows statistical significance (*p< 0.05). Intra-comparison in the observation and control groups shows statistical significance (**p< 0.05).

At the end of the study, compared with pre-treatment values, the absolute values of step length and height deviation in the control group decreased without significant difference (p> 0.05), but there was significant difference in the observation group (p< 0.05); the ratio of hip and knee flexion and extension mobility of bilateral lower limbs were significantly higher than pre-treatment in both two groups (p< 0.05). Moreover, significant differences were found in all the above factors between the two groups (p< 0.05) Table-V. No significant difference existed in the motion trajectory area and motion trajectory perimeter between the observation and control groups pre-treatment (p> 0.05), which were lower than pre-treatment values, and the observation group were lower than those in the control group, with statistically significant differences (p< 0.05). Table-VI.

**Table-V:** Comparison of Pre-and Post-Treatment Symmetry Parameters between the 2 Groups.

Indicators	Group	Pre-treatment	Post-treatment	p
Step length deviation(cm)	Observation	-3.100±6.477	-0.470±2.193	0.045**
	Control	-4.972±5.750	-4.032±3.168	0.343
	P	0.243	0.000^*^	
Step height deviation(cm)	Observation	2.267±1.946	0.670±1.768	0.000**
	Control	2.300±2.493	2.000±2.435	0.585
	P	0.954	0.018^*^	
Ratio of hip flexion and extension mobility of the affected and unaffected sides	Observation	0.714±0.152	0.841±0.100	0.000**
	Control	0.693±0.078	0.752±0.071	0.000**
	P	0.503	0.000^*^	
Ratio of knee flexion and extension mobility of the affected and unaffected sides	Observation	0.663±0.271	0.840±0.156	0.000**
	Control	0.645±0.138	0.743±0.098	0.000**
	P	0.737	0.006^*^	

***Note:*** Inter-comparison between the observation and control groups shows statistical significance (*p< 0.05). Intra-comparison in the observation and control groups shows statistical significance (**p< 0.05).

**Table-VI:** Comparison of Pre-and Post-Treatment Balance Indicators between the 2 Groups.

Indicator	Group	Pre-treatment	Post-treatment	p
Motion trajectory area(cm^2^)	Observation	429.053±210.171	228.830±106.391	0.000**
	Control	414.737±215.471	323.117±177.990	0.000**
	P	0.795	0.016^*^	
Motion trajectory perimeter(cm)	Observation	560.050±209.769	309.030±119.379	0.000**
	Control	546.997±202.387	395.733±153.438	0.000**
	P	0.807	0.018^*^	

***Note:*** Inter-comparison between the observation and control groups shows statistical significance (*p< 0.05). Intra-comparison in the observation and control groups shows statistical significance (**p< 0.05).

## DISCUSSION

The findings of this study demonstrate that after digital treadmill training, the active dorsiflexion angle of ankle joints, the mobility of hip and knee flexion and extension had significant improvement in the observation group compared to the control group. Literature indicates that treadmill training promotes neurovascular regeneration in the penumbra of cerebral ischemic by improving the basic fibroblast growth factor (bFGF)/caveolin-1 (Cav-1)/vascular endothelial growth factor(VEGF) pathway after ischemic stroke.^7^ For chronic stroke patients, the repetitive and intensive lower limb training provided by treadmills can help patients relearn sensorimotor skills and promote central nervous system remodeling. Therefore, treadmill training is effective in improving the walking abilities and balance function of stroke patients, regardless of the acute or chronic phase of the disease.^8^

This study found that not all step lengths on the affected side were larger than those on the unaffected side, which may be due to compensatory mechanisms in the affected lower limb, such as increased compensatory hip extension, forward trunk movement, and forward movement of the center of gravity. The study by Balaban et al^9^ suggests that there is variability in step length, which is influenced by various factors and may change as patients learn new tasks.

Meanwhile, another characteristic of hemiplegic gait is decreased walking speed. The study by Silver et al^10^ demonstrates that treadmill training can significantly improve the gait speed in hemiplegic patients, which is consistent with the findings of this study. Speed is related to many other spatial gait parameters, including step frequency, step length, duration of double support, duration of stance on the paretic and non-paretic sides, and stride cycle.^11^ Muscle strength of is an important factor, which is positively correlated with maximum walking speed^12^, especially hip flexor strength.^13^

Although about 80% of post-stoke patients regain their ability to walk, approximately half of them experience persistent gait asymmetry.^14^ Stroke survivors exhibit different patterns of gait asymmetry, however current research mainly focuses on the affected limb, overlooking the compensation of the unaffected limb. Therefore, both the affected and unaffected sides need to be fully considered in the rehabilitation training. It has been shown that step length deviation, the ratio of knee joint mobility and hip extension angle can better reflect the symmetry of lower limb joint movements.^15^ The findings of this study confirm that the step length and height deviation of both lower limbs were reduced post-treatment. The ratio of hip and knee joint flexion and extension mobility became closer to one, and the improvement in the observation group was more significant than in the control group.

Balance refers to the ability to adjust and maintain posture under movement or external force, which may determine the pattern of abnormal gait. Balance can be influenced by various factors, including restricted joint mobility, reduced muscle strength, changes in muscle tension, sensory deficits, abnormal posture, and cognitive impairments.^16^ During digital treadmill training, therapists assist patients to shift their body weight to the paralyzed side by controlling their trunk and pelvis from behind, reducing body sway to some extent.

Sensory input integration is crucial for maintaining balance, especially in cases of balance impairment visual feedback aids the restoration of proprioception. Research by Walker et al^17^ showed that providing visual feedback for trunk movement can help stroke patients reduce the sway of their body’s center of gravity, thereby enhancing dynamic balance control during walking. Specifically, digital treadmill training provides real-time visual feedback, allowing the affected limb to continuously correct body asymmetry by aligning with visual cues related to the body’s centerline, enabling precise weight shifting and enhancing balance.

In previous studies, the Berg Balance Scale (BBS) has often been applied in assessing the balance ability of patients. However, the scale is subject to significant subjective influence and may lead to floor and ceiling effects.^18^ In contrast, the balance instrument can objectively reflect the dynamic and static balance abilities of patients by using motion trajectory area and motion trajectory perimeter. The motion trajectory perimeter represents the total length of the center of gravity movement trajectory. A longer perimeter indicates a more obvious center of gravity swing and poorer stability.

At the same time, the motion trajectory area refers to the total area enclosed by the body’s center of gravity movement trajectory, reflecting the actual shape of the center of gravity movement and intuitively indicating the range of center of gravity movement, subtle postural adjustments, and the inherent reflex regulation ability of the spinal cord to posture.^19^ This study found that the motion trajectory area and motion trajectory perimeter for both groups of patients decreased significantly post-treatment, with the observation group showing a higher degree of improvement, and the difference was statistically significant(p< 0.05).

### Limitations:

A relatively small sample size was included in this study, which may lead to biased outcomes, and further validation is needed through studies with a larger sample size. Moreover, since the study was conducted for a relatively short period, further follow-up is needed to observe long-term efficacy.

## CONCLUSIONS

The application of digital treadmill training may be positive and effective in improving abnormal gait and enhancing the walking abilities of stroke patients. Due to the complex gait patterns of hemiplegic patients, specific issues should be analyzed when using digital treadmill training, and regular evaluation data should be used to tailor appropriate rehabilitation regimens for patients.

### Authors’ Contributions:

**YH** and **NW:** Carried out the studies, participated in collecting data, and drafted the manuscript, and are responsible and accountable for the accuracy or integrity of the work.

**SY: XY** and **YD:** Performed the statistical analysis and participated in its design. Critical Review.

All authors read and approved the final manuscript.
